# Oocytes With Smooth Endoplasmic Reticulum Aggregates Are Not Associated With Impaired Reproductive Outcomes: A Matched Retrospective Cohort Study

**DOI:** 10.3389/fendo.2021.688967

**Published:** 2021-08-26

**Authors:** Jian Xu, Li Yang, Zhi-Heng Chen, Min-Na Yin, Juan Chen, Ling Sun

**Affiliations:** Center for Reproductive Medicine, Guangzhou Women and Children’s Medical Center, Guangzhou Medical University, Guangzhou, China

**Keywords:** smooth endoplasmic reticulum aggregates, oocyte, cohort study, implantation rate, clinical pregnancy rate, live birth rate

## Abstract

**Objective:**

To investigate whether the reproductive outcomes of oocytes with smooth endoplasmic reticulum aggregates (SERa) are impaired.

**Methods:**

A total of 2893 intracytoplasmic sperm injection (ICSI) cycles were performed between January 2010 and December 2019 in our center. In 43 transfer cycles, transferred embryos were totally derived from SERa+ oocytes. Each of the 43 cycles was matched with a separate control subject from SERa- patient of the same age ( ± 1 year), embryo condition, main causes of infertility, type of protocols used for fresh or frozen embryo transfer cycles. The clinical pregnancy, implantation, ectopic pregnancy and live birth rate were compared between the two groups.

**Results:**

43 embryo transfer cycles from SERa- patient were matched to the 43 transferred cycles with pure SERa+ oocytes derived embryos. No significant difference was observed in clinical pregnancy rate (55.81% *vs*. 65.11%, *p*=0.5081), implantation rate (47.89% *vs*. 50.70%, *p*=0.8667) and live birth rate (48.84% *vs*. 55.81%, *p*=0.6659) between the SERa+ oocyte group and the matched group. No congenital birth defects were found in the two groups.

**Conclusion:**

Our results suggest that the implantation, clinical pregnancy, live birth and birth defects rate of embryos derived from oocytes with SERa are not impaired.

## Introduction

Aggregates of smooth endoplasmic reticulum (SERa) in the ooplasm is one of the cytoplasmic dysmorphisms of oocytes. These aggregations appear as round flat disks in the ooplasm corresponding to large tubular SER clusters surrounded by mitochondria (1).

Otsuki et al. firstly reported significantly lower pregnancy rates in SERa+ cycles and the transfer of embryos derived from SERa+ cycles resulted in Beckwith-Wiedmann syndrome (2). Subsequently, several studies have shown that significantly reduced pregnancy rates and a comparatively high number of congenital abnormalities in live born babies derived from SERa+ oocytes and/or cycles (1, 3, 4). Due to these adverse fetal outcomes, the Istanbul consensus in 2011 recommended not to use SERa+ oocytes (5).

After the consensus was published, more research focused on this field. However, the conclusions are inconsistent. The clinical pregnancy rate was comparable between SERa+ and SERa− cycles in the most of the published studies (1, 3, 6–13) except one literature which reported significant lower clinical pregnancy rates (2). Similar results were obtained when compared SERa+ oocytes with SERa- oocytes in SERa+ cycles, relatively lower pregnancy rate in SERa+ oocytes was observed in four studies (1, 6, 8, 13), but only in one study the difference reached statistical significance (13). Moreover, it was showed that there was no increase in congenital anomalies in embryos derived from SERa+ cycles and/or oocytes (6–9). Due to these discordant conclusions, reproductive endocrinologists and embryologists have varying attitudes when managing SERa+ oocytes. It was reported that only 14% of centers discarded SERa+ oocytes (14). As a result, the revised Vienna Alpha/ESHRE consensus reconsidered the recommendation and advised a case by case approach in 2017 (15).

However, most of the published literatures compared the clinical pregnancy outcomes between SERa+ and SERa− cycles (1–3, 6–13, 16, 17). It is not clear in these studies whether the transferred embryos were derived from SERa+ oocytes or not. Parts of these researches also reported the reproductive outcomes of embryos derived from SERa+ oocytes, but they were all directly compared with SERa- oocytes in the SERa+ cycle (6–9). However, the SERa- oocytes in SERa+ cycles maybe appear to be normal with light microscopy but on electron microscopy have been found to contain subtle, possibly pathological and small SERa (2).

Therefore, in order to clarify whether SERa have adverse effects on reproductive outcomes, direct comparison should be made between embryos derived from SERa+ oocytes in SERa+ cycles and embryos from SERa- cycles. Furthermore, it should be acknowledged that these studies are fraught with potential biases and confounders. Factors, such as maternal age, embryo conditions, main causes of infertility, type of protocol etc., were not matched in these studies, which could lead to inaccurate conclusions.

In this report, we conducted a matched, case-control study to determine whether the embryos derived from SERa+ oocytes were associated with negative reproductive outcomes.

## Materials and Methods

### Study Design and Study Participants

All women undergoing intracytoplasmic sperm injection (ICSI) cycles that did not involve pre-implantation genetic testing (PGT) in the Centre for Reproductive Medicine, Guangzhou Women and Children’s Hospital between January 2010 and December 2019 were included in the study. Since the presence of SERa cannot be observed on the day of insemination, patients undergoing conventional IVF and split IVF-ICSI were excluded from the study. The study was approved by the Independent Ethics Committee of Guangzhou Women and Children’s Hospital.

SERa+ oocytes were defined as those oocytes where one or more SERa were visible with an inverted microscope after denudation just prior to ICSI. A SERa+ cycle indicates that at least one SERa+ oocyte is observed among the cohort. The SERa- cycles had morphologically normal oocytes. When all the cycles are SERa-, the patient is defined as a SERa- patient.

Both fresh and frozen embryo transfer (ET) cycles were included in the study. The reproductive outcomes were only analyzed in transfer cycles where transferred embryos were totally derived from SERa+ oocytes.

### Patient Treatment

Standard controlled ovarian stimulation protocols were used. Briefly, the patients underwent either GnRH agonist or GnRH antagonist protocol for ovulation induction. The ovarian stimulation was performed using recombinant FSH (Gonal-F, Merck-Serono, Geneva, Switzerland) in combination with GnRH antagonist (Cetrorelix Acetate, Merck-Serono, Geneva, Switzerland) or agonist (Triptorelin Acetate, Ipsen Pharma Biotech, France). The initial doses were based on antral follicle counts, female age, and basal FSH. The subsequent doses were adjusted according to follicle growth and serum estradiol levels. HCG or GnRH agonist was administered when at least three leading follicles reached a mean diameter ≥ 18 mm. Transvaginal oocyte retrieval was scheduled 34–38 h later.

### Embryo Culture and Morphological Assessment

Conventional ICSI was performed 4–6 h after the oocyte retrieval. At the time of ICSI, each oocyte was evaluated for the presence of cytoplasmic abnormalities using an inverted microscope and data were recorded. The large SERa present in the cytoplasm of MII resembles a vacuole but can be easily distinguished from a vacuole since it is not fluid filled and not separated from the rest of the cytoplasm by a membrane (18). During the ICSI procedure, we carefully avoided rupturing the aggregates. The fertilization check was done at 16–18 h after insemination. Zygotes were cultured individually in G1 (Vitrolife, Sweden) media under 6% CO_2_, 37°C until days three. Embryos with extended culture were transferred on day three to G2 (Vitrolife, Sweden) media and cultured individually under the same conditions until day five or six. Cleavage stage embryos were assessed for cell stage, percent fragmentation, multinucleation, and blastomere symmetry according to ESHRE/ALPHA consensus (5). Good quality cleavage-stage embryos were defined as those with the following characteristics: 7-8 cells on day 3, <10% fragmentation, symmetric blastomeres, and the absence of multinucleated blastomeres. A good quality blastocyst was defined according to modified Gardner and Schoolcraft grading (19).

### Embryo Selection for Cryopreservation or Transfer

When selecting embryos for cryopreservation or transfer, we mainly based on the morphological embryo scoring. The presence of SERa in corresponding metaphase II oocytes was not taken into account. Embryos were frozen according to the vitrification protocol and thawed using a standard protocol as instruction described (Jieying thawing medium, Jieying Laboratory Inc., Canada). The embryo transfers were done with ultrasound guidance by using a standard embryo transfer catheter (Wallace, Smith medical, Mexico).

### Matched Case-Control Study

In 43 transfer cycles, including 13 fresh transfer cycles and 30 frozen transfer cycles, the transferred embryos were totally derived from SERa+ oocytes. The matched group was drawn from all ICSI cycles from SERa- patients. Matching was accomplished by the following criteria: [1] age( ± 1 year); [2] embryo condition (embryo stage, number and quality) [3] main causes of infertility (male factor, tubal factor, endometriosis et. al); [4] type of protocol used for controlled ovarian hyperstimulation (COH) in fresh embryo transfer or endometrium preparation protocol in frozen embryo transfer; [5] infertile years ( ± 2 year); [6] number of transfer cycles; [7] embryo transfer date ( ± 1 year); [8] embryo transfer physician. We required exact matching for criteria 1–4; for criteria 5–8, we attempted matching as closely as possible and in most cases were able to match at least two of these criteria. To reduce the introduction of potential bias, researchers were blinded to reproductive outcomes during the matching process. If multiple patients fit the criteria, one was chosen at random.

### Clinical Follow-Up

The implantation rate was calculated as the number of gestational sacs divided by the number of transferred embryos. Clinical pregnancy was defined as the presence of an intrauterine gestational sac with a yolk sac, a fetal pole, and fetal heart pulsations. Ectopic pregnancies were diagnosed by ultrasound or by laparoscopic visualization of an extrauterine gestational sac or by the absence of an intrauterine gestational sac and increasing β-hCG levels following the failure of suction D&C to reveal products of conception. Live birth was defined as the birth of a live infant at ≥28 weeks of gestation.

### Statistical Analysis

Data are presented as the mean ± standard deviation. Statistical comparisons of 2 experimental groups were evaluated by 2-tailed Student t test if data distribution passed the normality test. The Mann-Whitney U test was used for comparison if data distribution failed the normality test. A chi-square test or Fisher’s exact test to compare fertilization, pregnancy and live birth rate etc. *P* value <0.05 was considered statistically significant.

## Results

A total of 2893 ICSI cycles were performed between January 2010 and December 2019 in our center. Among them, 482 were SERa+ cycles, the incidence rate was 16.7%. The baseline characteristics, stimulation protocols and outcomes of SERa+ cycles and SERa- cycles are shown in **Table 1**. No significant difference was observed in BMI (21.4 ± 2.7 *vs*. 21.6 ± 3.0), stimulation protocols and total gonadotrophins dose (2090.7 ± 918.7 *vs*. 2043.8 ± 943.9) between the two groups. Compared with SERa-cycles, significant younger age (33.5 ± 5.4 *vs*. 34.5 ± 5.6), more stimulation days (9.3 ± 2.5 *vs*. 8.9 ± 2.8), higher estradiol levels at hCG-trigger (9973.3 ± 5403.3 *vs*. 8662.2 ± 5477.4), and more mean number of retrieved oocytes (11.5 ± 7.9 *vs*. 8.8 ± 7.1) and MII oocytes (9.9 ± 6.7 *vs*. 7.4 ± 5.8) were found in SERa+ cycles.

**Table 1:** Baseline characteristics, stimulation protocols and outcomes of SERa+* cycles and SERa- cycles.

Item	SERa+ cycles (n = 482)	SERa- cycles (n = 2411)	P
Age (years)	33.5 ± 5.4	34.5 ± 5.6	<0.001
BMI (kg/m^2^)	21.4 ± 2.7	21.6 ± 3.0	0.1377
Stimulation protocols			0.9110
antagonist protocol	189 (39.2%)	952 (39.5%)	/
agonist protocol	293 (60.8%)	1459 (60.5%)	/
Duration of stimulation (days)	9.3 ± 2.5	8.9 ± 2.8	<0.001
Total gonadotrophins dose	2090.7 ± 918.7	2043.8 ± 943.9	0.3143
Estradiol levels at hCG-trigger (pmol/L)	9973.3 ± 5403.3	8662.2 ± 5477.4	<0.001
Mean no. of retrieved oocytes	11.5 ± 7.9	8.8 ± 7.1	<0.001
Mean no. of MII oocytes	9.9 ± 6.7	7.4 ± 5.8	<0.001

*SERa, aggregates of smooth endoplasmic reticulum.

In the 482 SERa+ cycles, 43 embryo transfer cycles were exclusively derived from SERa+ oocytes, including 13 fresh transfer cycles and 30 frozen transfer cycles. Totally 71 embryos, including 39 good quality embryos, were transferred. 43 embryo transfer cycles from SERa- patients were matched to the pure SERa+ oocyte group (1:1).

The cycle characteristics of pure SERa+ oocyte group and matched group are presented in **Table 2**. There were no significant differences between the two groups in terms of age, body mass index (BMI), duration of infertility, mean number of transfer cycles, endometrial thickness, stimulation protocols etc. The embryological outcomes of the two groups are presented in **Table 3**. The normal fertilization rate (82.75% *vs*. 86.05%), cleavage rate (99.09% *vs*. 97.80%) and D3 good embryo quality rate (33.84% *vs*. 32.69%) were all comparable between SER+ oocyte group and the matched group. **Table 4** presents the main reproductive outcomes of the two groups. No significant difference was observed in clinical pregnancy rate (55.81% *vs*. 65.11%), implantation rate (47.89% *vs*. 50.70%), ectopic pregnancy rate (4.17% *vs*. 0%), miscarriage rate (8.33% *vs*. 14.29%), live birth rate (48.84% *vs*. 55.81%), gestational week at birth (37.9 ± 1.7 *vs*. 37.8 ± 1.8), or birth weight (2.9 ± 0.6 *vs*. 2.8 ± 0.6) between the SERa+ oocyte group and the matched group. From these 43 transfers in SERa+ oocyte group, 21 live births were obtained and resulted in the birth of 28 healthy infants (7 twin pregnancy). No congenital anomalies were found in the two groups.

**Table 2:** Baseline characteristics of SERa+* oocyte group and matched group.

Item	SERa+ oocyte group (n = 43)	Matched group (n = 43)	*P*
Age (years)	32.5 ± 6.1	32.5 ± 6.1	0.9718
BMI (kg/m^2^)	21.4 ± 3.0	21.2 ± 2.8	0.7478
History of infertility (years)	4.6 ± 3.3	3.9 ± 2.3	0.2605
Mean no. of transfer cycles	1.3 ± 0.5	1.3 ± 0.5	0.6802
Day 3 serum FSH (IU/mL)	6.0 ± 1.3	6.0 ± 1.6	0.8650
Stimulation protocols			1.0000
antagonist protocol	15(34.9%)	15 (34.9%)	
agonist protocol	28(65.1%)	28 (65.1%)	
Fresh or frozen embryo transfer			1.0000
Cycles of fresh embryos transfer	13 (30.23%)	13 (30.23%)	
Cycles of frozen embryos transfer	30 (69.77%)	30 (69.77%)	
Stage of embryos transfer			1.0000
Cycles of cleavage embryos transfer	36 (83.72%)	36 (83.72%)	
Cycles of blastocysts transfer	7 (16.28%)	7 (16.28%)	
Endometrium thickness (mm)	10.3 ± 2.4	9.7 ± 1.7	0.1585

*SERa, aggregates of smooth endoplasmic reticulum.

**Table 3:** Embryological outcomes of SERa+* oocyte group and the matched group.

Item	SERa+ oocyte group (n = 43)	Matched group (n = 43)	P
Mean oocytes	10.6 ± 6.9	11.6 ± 6.8	0.2411
MII oocyte rate	400/456 (87.72%)	423/501 (84.43%)	0.1432
Normal fertilization rate	331/400 (82.75%)	364/423 (86.05%)	0.1914
Cleavage rate	328/331 (99.09%)	356/364 (97.80%)	0.1731
D3 good quality embryo rate	112/331 (33.84%)	119/364 (32.69%)	0.7490

*SERa, aggregates of smooth endoplasmic reticulum.

**Table 4:** Reproductive outcomes of SERa+* oocyte group and the matched group.

Item	SERa+ oocyte group (n = 43)	Matched group (n = 43)	*P*
Clinical pregnancy rate	24/43 (55.81%)	28/43 (65.11%)	0.5081
Implantation rate	34/71 (47.89%)	36/71 (50.70%)	0.8667
Ectopic pregnancy rate	1/24 (4.17%)	0/28 (0%)	0.2754
Miscarriage rate	2/24 (8.33%)	4/28 (14.29%)	0.4485
Live birth rate	21/43 (48.84%)	24/43 (55.81%)	0.6659
Gestational week	37.9 ± 1.7	37.8 ± 1.8	0.7531
Birth weight (kg)	2.9 ± 0.6	2.8 ± 0.6	0.4789

*SERa, aggregates of smooth endoplasmic reticulum.

## Discussion

The occurrence rates of SERa reported in previous researches are different, ranging from 5.4% to 23.1% (20). Consistent with these studies, the incidence rate of SERa+ cycles was 16.7% in our center. The mechanism underlying SERa formation remains currently unknown and data about the risk of recurrence of SERa oocyte are still unclear. Similar to those of previous studies, our results showed that prolonged stimulation days, higher estradiol levels at hCG-trigger, and more mean number of retrieved oocytes were observed in SERa+ cycles (2, 3, 6, 11).

Since Otsuki et al. firstly reported significantly lower pregnancy rate and fetal anomalies derived from SERa+ oocytes (2), more and more researchers have paid attention to the existence of SERa. As data about the clinical outcomes of SERa+ oocytes are discordant, reproductive endocrinologists and embryologists have varying attitudes when managing SERa+ oocytes.

Transfer of embryos originating from SERa+ oocytes was not avoided in our center. Previous investigation also showed that only 14% of centers discarded SERa+ oocytes and 43% of centers that did not discard oocytes followed up neonatal data (14). For this reason, three types of transfer could be divided in SERa+ cycles: embryo transfer with SERa+ oocytes only, SERa- oocytes only and mixed SERa oocytes. If the presence of SERa in the cytoplasm of oocytes has a negative impact on clinical and neonatal outcome, the reproductive outcomes of the SERa+ oocytes derived embryo transfer cycle are most likely to be affected.

In our study, there are 43 transfer cycles in which transferred embryos were totally derived from SERa+ oocytes. More than half of the embryos were good quality embryos (39/71) according to our embryo scoring criteria. This may explain the high pregnancy rate (55.81%) and live birth rate (48.84%) in our SERa+ oocyte group. 21 out of the 43 transfer cycles had alternative embryos, but embryo scoring was better in the SERa+ oocytes derived embryos. In the left 22 cycles in which no alternative embryos existed, the mean age (34.9 ± 7.0) was older, mean number of retrieved oocytes (7.0 ± 5.8) was relatively small. The acceptable clinical pregnancy rate (45.5%) and live birth rate (36.4%) in the 22 aged cycles also indicated that the clinical outcomes of SERa+ embryos are not impacted when there is no other alternative in embryo selection.

To minimize the impact of the clinical heterogeneity observed between patients, we used multiple matching criteria. We attempted in particular to match for age, embryo condition, main causes of infertility, type of protocol used for COH in fresh embryo transfer or endometrium prepare protocol in frozen embryo transfer, which are known to affect IVF success. In our study, 43 transfer cycles with pure SERa+ oocyte derived embryos were included and compared with matched controls. This suggests that SERa+ oocytes are not associated with both decreased clinical pregnancy rate, implantation rate, gestational week, birth weight, live birth rate, and increased miscarriage rate and birth defect rate.

As we mention above, if the presence of SERa in the cytoplasm of oocytes has a negative impact on clinical and neonatal outcome, the reproductive outcomes of the SERa+ embryo transfer cycle are most likely to be affected. After finding that the reproductive outcomes of SERa+ embryos were totally normal, we decided not to continue other subgroup analysis.

Gurunath et al. reported that a gradual reduction in live birth rates was observed when the percentage of oocytes containing SERa increased and the group containing >50% of oocytes with SERa demonstrated no live births (16). However, in our data, two patients with all oocytes presenting SERa delivered three healthy newborn (one twin delivery). In their study, embryos derived from SERa+ oocytes were not preferred for transfer and they were utilized only when there were no other embryos suitable for transfer. It is very probable that poor embryo quality but not the increased percentage of oocytes containing SERa caused the reduction in live birth rates.

Whether SERa increase birth defects has always attracted extensive attention. Based on previously reported cases of Beckwith-Wiedemann syndrome (2), diaphragmatic hernia (3), and multiple malformations (4) after transfer of embryos derived from SERa+ oocytes, the presence of SERa is reportedly associated with an increased risk of baby malformations. This is also the major reason why the Istanbul Consensus Workshop advised against their use in ICSI (5). In a recent review, after summarizing 9 publications (including case reports), a total of 48 healthy newborns and four babies with perinatal complications after transfer of embryos exclusively derived from SERa+ oocytes were reported. It was the author’s conclusion that the ethical practice and the best care for patients favored fertilizing both SERa+ and SERa- oocytes and prioritizing embryos derived from SERa- oocytes (21). Our study reports the delivery of the highest number healthy babies after transfer with embryos originating from SERa+ oocytes analyzed so far. No major malformations were seen in any of the 28 babies.

Some limitations of our research should be noted. The nature of SERa makes it a retrospective study, which by nature cannot exclude selection bias. Furthermore, SERa can be classified on the basis of its size: large, medium and small aggregates (2). In most cases, including our study, the SERa is observed under light microscope. The small size SERa and perhaps some medium size SERa cannot be seen under light microscope and their incidence is still unknown. A bias might have been introduced because we can only observe the large and medium SERa under light microscopy. Moreover, to make our results more accurate, strict inclusion criteria were carried out, which inevitably resulting in a small sample size, and the results should be interpreted with caution.

## Conclusion

The implantation, clinical pregnancy, live birth and birth defects rate of embryos derived from oocytes with SERa are not impaired.

## Data Availability Statement

The datasets generated for this study are available on request to the corresponding author. Requests to access the datasets should be directed to LS, sunling6299@163.com.

## Ethics Statement

The studies involving human participants were reviewed and approved by Independent Ethics Committee of Guangzhou Women and Children’s Hospital. Written informed consent for participation was not required for this study in accordance with the national legislation and the institutional requirements.

## Author Contributions

LS was responsible for the conception and design of the study, interpretation of data, revised the article critically for important intellectual content, and approved the final draft for publication. JX and LY contributed to collect the data, analysis and interpretation of data; draft and revise the whole article. Z-HC, M-NY, and JC contributed to collecting the data, drafting and revising the article for important intellectual content. All authors contributed to the article and approved the submitted version.

## Funding

This study is supported by the National Natural Science Foundation of China (81800110).

## Conflict of Interest

The authors declare that the research was conducted in the absence of any commercial or financial relationships that could be construed as a potential conflict of interest.

## Publisher’s Note

All claims expressed in this article are solely those of the authors and do not necessarily represent those of their affiliated organizations, or those of the publisher, the editors and the reviewers. Any product that may be evaluated in this article, or claim that may be made by its manufacturer, is not guaranteed or endorsed by the publisher.
